# Dolutegravir/Lamivudine versus Tenofovir Alafenamide/Emtricitabine/Bictegravir as a Switch Strategy in a Real-Life Cohort of Virogically Suppressed People Living with HIV

**DOI:** 10.3390/jcm12247759

**Published:** 2023-12-18

**Authors:** Giuseppe Vittorio De Socio, Sara Tordi, Debora Altobelli, Anna Gidari, Anastasia Zoffoli, Daniela Francisci

**Affiliations:** Department of Medicine, Clinic of Infectious Diseases, “Santa Maria della Misericordia” Hospital, University of Perugia, 06129 Perugia, Italy; debora.altobelli@ospedale.perugia.it (D.A.); anna.gidari@ospedale.perugia.it (A.G.); anastasia.zoffoli@ospedale.perugia.it (A.Z.); daniela.francisci@unipg.it (D.F.)

**Keywords:** HIV, dolutegravir, bictegravir, virological failure, safety, toxicity

## Abstract

Background: The aim of the study is to evaluate the effectiveness, safety, and tolerability of a two-drug regimen (2-DR) dolutegravir/lamivudine (DTG/3TC) versus a three-drug regimen (3-DR) tenofovir alafenamide/emtricitabine/bictegravir (TAF/FTC/BIC) in a real-life cohort of HIV-1 virologically suppressed treatment-experienced (TE) people living with HIV (PLWH). Methods: This was a single-center, retrospective, observational study analyzing adult TE PLWH who started the 2-DR or 3-DR between January 2018 and January 2023. All PLWH with a viral load (VL) <50 copies/mL at the time of switching, and a follow-up of more than 6 months or interruption of treatment at any time, were included. Results: A total of 324 PLWH were included; of these, 110 (34%) were on the 2-DR and 214 (66%) were on the 3-DR. Most patients remained on therapy in both groups (93.6% 2-DR versus 90.2% 3-DR) and, at the last control, 99.1% achieved VL < 50 copies/mL with the 2-DR versus 97.2% with the 3-DR (*p* = 0.260). No virological failures occurred in either group. Adverse events occurred in a few cases: four (3.6%) in the 2-DR group and five (2.3%) in the 3-DR group (*p* = 0.500). The median follow-up-time was 19.6 months for the 2-DR and 27.5 months for the 3-DR. Conclusion: Our study shows a similar effectiveness and safety profile in virologically suppressed PLWH switching to DTG/3TC or TAF/FTC/BIC.

## 1. Introduction

The second-generation integrase inhibitors (INIs) dolutegravir (DTG) and bictegravir (BIC) play an important role as an initial or switch strategy due to their virological efficacy, high genetic barrier, low drug–drug interactions, good tolerability and safety, and availability as fixed-dose combination (FDC) single-tablet regimens (STR) [1,2,3,4,5,6,7,8,9,10,11,12,13,14].

Many studies have shown the therapeutic effect of two-drug regimens based on DTG/3TC; over the years, this therapeutic effect has not been inferior to that of three-drug regimens. The phase-III TANGO randomized clinical trial (RCT) showed the virological efficacy of switching to a dolutegravir/lamivudine (DTG/3TC) combination, which was not inferior to the continuation of the TAF-based regimen at 144 weeks [15]. In the GEMINI trials, DTG/ 3TC and DTG plus tenofovir alafenamide/emtricitabine (FTC/TAF) showed similar rapid decreases in plasma viral load, regardless of the baseline viral load [12]. Similarly, BIC is one of a new generation of INIs; available as a three-drug FDC with emtricitabine/tenofovir alafenamide, it has shown high efficacy in large phase-III randomized clinical trials involving either naïve or already virologically suppressed people living with HIV (PLWH) [6,7].

The three-drug regimen (3-DR) tenofovir alafenamide/emtricitabine/bictegravir (TAF/FTC/BIC) and the two-drug regimen (2-DR) dolutegravir/lamivudine (DTG/3TC) are recommended for most patients as an initial treatment for naïve patients and as a switch strategy for treatment-experienced (TE) PLWH [1,2,3,4,5].

Parallel to the widespread use of second-generation INIs, a paradigm shift from 3-DRs to 2-DRs began to take place in clinical practice. One of the main goals of 2-DRs, e.g., DTG/3TC, is maintaining an excellent virological efficacy and high genetic barrier [11,12,13,14]. Furthermore, 2-DRs reduce the potential risk of short- and long-term toxicities, mainly related to the adverse effects (AEs) of the NRTI backbone on renal function and bone mineral density [14,15,16].

Several studies have explored the effectiveness, safety, and tolerability of DTG/3TC [17,18,19,20,21,22,23,24,25,26,27,28] or TAF/FTC/BIC [29,30,31,32,33,34] in real-world cohorts of virologically suppressed PLWH. However, to date, there are few comparison data on the effectiveness, tolerability, and safety of these regimens as a switch strategy in clinical practice [35,36,37,38] and a lack of comparative randomized studies. Such works have clinical relevance as the enrolled patients typically have a more complex therapeutic and clinical history than those in RCTs [6,7,11,12,15,16,39,40,41].

Our primary aim is to compare the virological effectiveness of the 2-DR DTG/3TC versus the 3-DR TAF/FTC/BIC in a real-life cohort of HIV-1 virologically suppressed TE PLWH. Our secondary aim is to evaluate safety and tolerability, regimen discontinuation for any reason, and regimen discontinuation due to AEs and virological failures.

## 2. Materials and Methods

We carried out a single-center, retrospective, observational study analyzing adult TE PLWH switching to the 2-DR STR DTG/3TC or the 3-DR STR TAF/FTC/BIC between January 2018 and January 2023 at the Infectious Diseases Clinic, Santa Maria della Misericordia University Hospital, University of Perugia, Italy, according to the physician’s decision. In our center, antiretroviral therapy is prescribed by a total of five physicians, most of whom have more than 20 years of experience in HIV care. PLWH were identified by examining electronic medical prescription records for the issue.

Eligible PLWH had an age ≥ 18 years, a viral load (VL) < 50 copies/mL at the baseline (time of switch to one of the two regimens analyzed), no evidence of resistance mutations to INIs or previous failure to INIs, and more than 6 months of follow-up or treatment interruption at any time. We excluded PLWH lost to follow-up. The follow-up time was defined as the period from the switch to TAF/FTC/BIC or DTG/3TC to the last visit. We recorded demographic and clinical characteristics at the baseline (age, sex at birth, nationality, sexual orientation, comorbidities, body mass index (BMI), levels of hepatitis C virus antibodies, HbsAg positivity, date of diagnosis, CD4 cell count, and VL at HIV presentation and at the last control), date and reason of discontinuation, and the lipid profile at the baseline and at the last control after the switch.

The biochemistry serum panel, plasma HIV-RNA, and CD4 cell count were tested by the laboratory of our hospital. HIV RNA was quantitated by polymerase chain reaction using a Roche Cobas HIV-1 5800 System (Roche Molecular Systems, Pleasanton, CA, USA) with a limit detection of 20 copies/mL. For any PLWH enrolled, existing routine clinical genotypic resistance tests (Sanger method) were evaluated according to the Stanford HIV Drug Resistance Database (Stanford HIVdb) [42], and cumulative data were considered for HIV drug resistance (DR). In our analysis, we defined no resistance as the absence of major resistance mutation to any class of the principal antiretroviral class drugs, nucleoside reverse transcriptase inhibitors (NRTIs), non-nucleoside reverse transcriptase inhibitors (NNRTIs), protease inhibitors (PIs), and INIs.

The primary endpoint was the maintenance of VL < 50 copies/mL. Secondary endpoints included safety and tolerability, as well as treatment discontinuation (TD) for any cause. Subsequently, we classified the reasons for TD as adverse events (AEs), patient’s death, virological failure, and switch or simplification to another regimen according to the provider’s choice. AEs were further classified as neurological toxicity (headache, sleep disturbances, dizziness, etc.), skin manifestations (rash, hypersensitivity reaction, etc.), gastrointestinal toxicity (nausea, vomiting, etc.), renal toxicity (declining estimated glomerular filtration rate, proteinuria, etc.), or musculoskeletal (myalgia, arthralgia, creatinine phosphokinase (CPK) elevations, etc.). A single value of detectable HIV RNA between 50 and 200 copies/mL during the follow-up period was defined as a blip. A detectable HIV RNA between 50 and 200 copies in two or more controls was defined as low-level viremia (LLV). VF was defined as the presence of at least two VL > 200 copies/mL or one VL > 1000 copies/mL after the exclusion of adherence failure [1].

Furthermore, we evaluated the median change in CD4 cell count (delta) from the baseline to the last control.

Categorical variables are presented using frequency tables, and continuous variables are presented in terms of median with interquartile range (IQR). Differences between groups of several characteristics were analyzed using the Mann–Whitney U test or χ2 test, as appropriate. Continuous variables were tested to detect substantial deviations from normality by computing the Kolmogorov–Smirnov Z test (the assumption of satisfactory normal distribution was met for all the examined variables) to determine whether the numerical variables fit the assumption of normality. Continuous variables were tested by Student’s *t*-test or Mann–Whitney U test as appropriate and a paired samples *t*-test was used for two repeated measurements. Kaplan–Meier analysis was performed to identify differences in discontinuation rate between the two regimens, after excluding discontinuation due to simplification to a 2-DR regimen at the physician’s discretion. Statistical analyses were performed with SPSS 20.0 (SPSS, Chicago, IL, USA).

The study was approved by the local Ethics Committee (protocol number CER 4551/23) and all patients at admission had signed for permission to use their data.

## 3. Results

**Figure 1:**
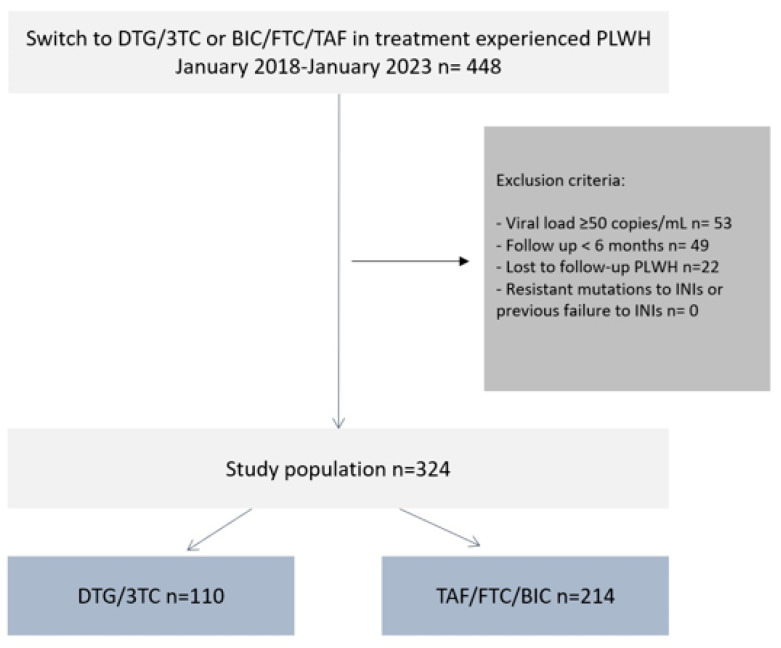
Flow-chart of the study.

Between January 2018 and January 2023, 448 adult TE PLWH switched to the 2-DR STR DTG/3TC or 3-DR STR TAF/FTC/BIC. According to the exclusion criteria, a total of 324 PLWH were included in the analysis, as reported in Figure 1. The baseline characteristics are shown in Table 1.

Of these, 110 (34%) were on the 2-DR and 214 (66%) were on the 3-DR. The median age of the 2-DR cohort was 56.0 years [IQR 44.0–63.0] and for the 3-DR cohort 56.5 years [IQR 48.0–61.5] (*p* = 0.653); there were 82 (74.5%) males in the 2-DR group and 172 (80.4%) in the 3-DR group.

As reported in Table 1, comorbidities were significantly more represented in the 2-DR group (median number of comorbidities: two [IQR 1–5] in the 2-DR versus one [IQR 0–2] in the 3-DR), specifically: hypertension, cardiovascular diseases, dyslipidemia, and chronic kidney disease. PLWH in the 3-DR were significantly more coinfected with HCV and HBV and showed lower baseline CD4 levels (585 cells/μL on 3-DR versus 781.5 cells/μL on 2-DR, *p* < 0.0001) and CD4 nadir (214 cells/μL on 3-DR versus 297 cells/μL on 2-DR, *p* = 0.015), and a less favorable genotype resistance test.

In both groups, PLWH had a long history (132 months 2-DR versus 120 3-DR) of combination antiretroviral therapy (cART) due to previous INI-based regimens, followed by TAF-based regimens for the 3-DR and ABC-based regimens for the 2-DR. The median time of virological suppression prior to switching was 98.1 months [IQR 57–151] in the 2-DR versus 80.5 [IQR 39.5–138.7] in the 3-DR (*p* = 0.035). The median follow-up was 19.6 months [IQR 14.2–26.4] in the 2-DR cohort and 27.5 months [IQR 15.3–32.6] in the 3-DR cohort (*p* = 0.001).

### 3.1. Treatment Discontinuation and Adverse Events

Overall, the discontinuation rate was very low as most PLWH remained on therapy in both groups (93.6% 2-DR versus 90.2% 3-DR).

PLWH on the 3-DR showed more treatment discontinuations, but these were mainly due to the physician’s decision to simplify to the 2-DR (11 cases, 5.1%). Three PLWH (1.4%) died in the 3-DR group; the causes of death were not associated with HIV or cART (one due to hemorrhagic and septic shock, one due to suicide, and one due a fatal event not characterized). Among the episodes of TD, four PLWH (3.6%) switched due to AEs in the 2-DR group and five (2.3%) in the 3-DR group (*p* = 0.500) (Table 2).

**Figure 2:**
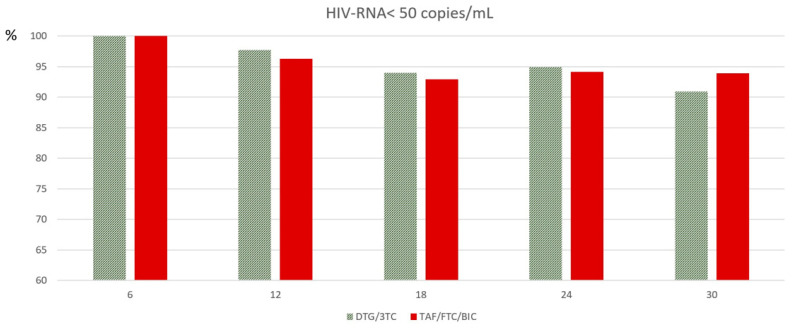
DGT/3TC and TAF/FTC/BIC showed a similar rate of maintenance HIV-RNA < 50 copies/mL for PLWH on therapy at controls between 6 and 30 months of follow-up (*p* ns at all controls).

**Figure 3:**
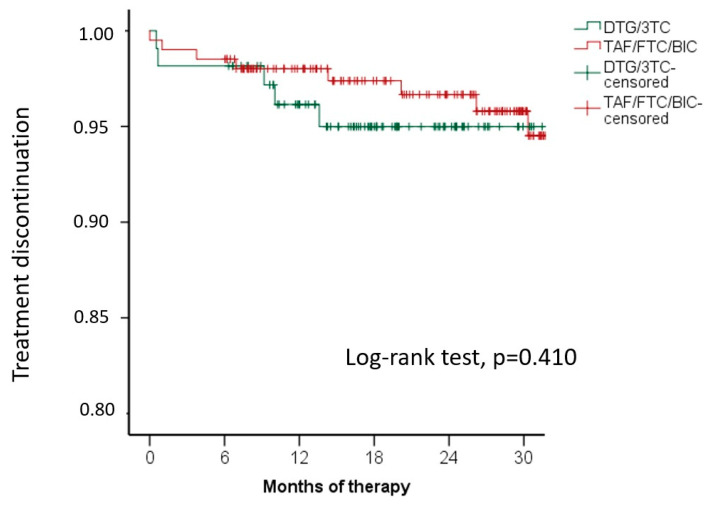
Kaplan-Meier plots showing the estimates probability of treatment discontinuation (Survival analysis); DGT/3TC and TAF/FTC/BIC showed a similar discontinuation rate for any reason (12 cases of discontinuation due to simplification to a 2-DR were not included in the survival analysis).

As shown in Figure 2, maintenance of HIV-RNA < 50 copies/mL at 6, 12, 18, 24, and 30 months was not different between the study groups (*p* = ns at any controls); the discontinuation rate was not different between the 2-DR and the 3-DR (Figure 3). The comparison between groups was the same after the exclusion (log-rank test, *p* = 0.410) or inclusion (log-rank test, *p* = 0.897) of 12 cases of discontinuation due to simplification to a 2-DR regimen. The reasons for TD due to AEs were mainly musculoskeletal, i.e., myalgia and CPK elevations in both groups, followed by neurological toxicity. Interestingly, renal toxicities concerned only one patient in the 3-DR group. No symptoms were life-threatening or led to hospitalization.

### 3.2. Effectiveness

**Figure 4:**
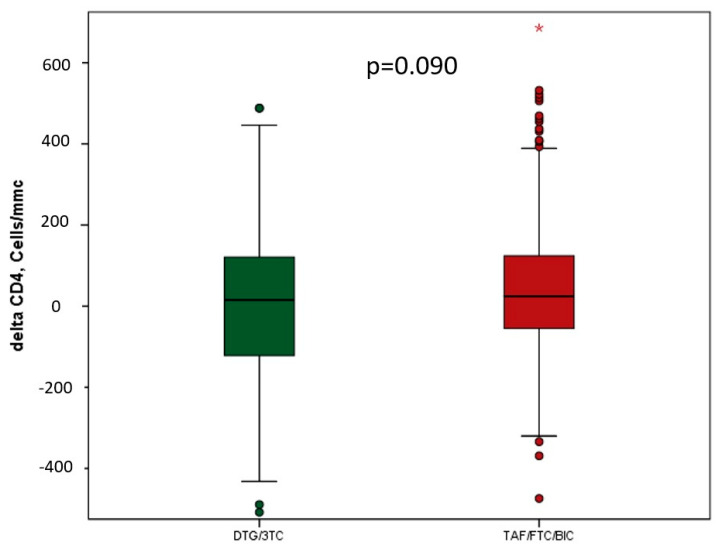
Delta CD4 cells count (last control—baseline) in PLWH treated with DTG/3TC or TAF/FTC/BIC (paired samples *t*-test). The asterisk (*) represents out of limit values.

Overall, at the last control, 99.1% achieved VL < 50 copies/mL in the 2-DR versus 97.2% in the 3-DR (*p* = 0.260) (difference: 1.9%; 95% CI 0.37 to 2.64). The results of VL < 50 copies/mL were similar between groups at any interval (every 6 months), as shown in Figure 2. Discontinuation due to low-level viremia (LLV) occurred in 1.8% of individuals on the 2-DR versus 0.9% on the 3-DR (*p* = 0.495) and blip in 4.5% of individuals on the 2-DR versus 11.8% on the 3-DR (*p* = 0.033). No virological failure was observed in either group during the follow-up period, and a single value of HIV-RNA >200 copies/mL was reported in only one person in the TAF/FTC/BIC group due to treatment adherence. The CD4 cell count improved in both groups without a significant difference (Figure 4).

### 3.3. Metabolic Profile

The lipid profile improved in both groups without a significant difference between the 2-DR and 3-DR (Table 2). In particular, no significant changes were observed in any evaluated parameters (total cholesterol, low-density lipoprotein cholesterol, and triglycerides) during the follow-up period. Similarly, the median BMI did not show a change from the baseline.

## 4. Discussion

The study showed that PLWH from a real-life cohort, who switched to TAF/FTC/BIC or DTG/3TC, had high rates of maintaining virological suppression at the last control (99.1% in 2-DR versus 97.2% in 3-DR), as well as every six months between 6 and 30 months; this highlights the good efficacy of second-generation INI-based regimens in this setting.

The differences in baseline characteristics are related to the real-life approach taken by the physicians. The 2-DR regimen was preferred in the presence of comorbidities and 3-DR in the presence of more advanced HIV disease (low CD4 nadir and CDC C stage); this complicates the direct comparison of efficacy among real-life treatment-experienced PLWH cohorts. Our findings support the hypothesis of Rocabert et al. that clinicians may prefer to prescribe DTG/3TC in a more conservative way than TAF/FTC/BIC; in a large Spanish real-life cohort, DTG/3TC was generally preferred in older people with a better immunological and virological status at the time of switching [36].

The viral suppression achieved by both regimens is generally consistent across previous real-world studies and compared to those reported in clinical trials [6,7,8,9,10,11,12,13,14,15,16,17,18,19,20,21,22,23,24,25,26,27,28,29,30,31,32,33,34,35,36,37,38]. Our findings are consistent with Knobel et al., who conducted a real-world study comparing DTG/3TC and TAF/FTC/BIC as a switching strategy in virologically suppressed PLWH. They described a rate of maintenance of HIVRNA < 50 copies/mL of 94.4% and 96.1% in the DTG/3TC and TAF/FTC/BIC groups, respectively [38]. Similarly, Gan et al. in a real-world cohort documented a rate of viral suppression at 48 weeks of 95.9% for DTG/3TC and 95.6% for TAF/FTC/BIC [37]. Punekar et al. conducted a meta-analysis with the objective of estimating the effectiveness and tolerability of 2-DRs (DTG-based regimens) in clinical practice. The authors found viral suppression in the treatment analysis (VSOT) of 98.8% and 98.4% for DTG/3TC at 48 and 96 weeks, respectively [26]. Another meta-analysis by Patel et al. confirmed the same high rates of the effectiveness and safety of DTG/3TC in several real-life studies, namely virological suppression from 97% to 100% and 92–100% at 48 weeks and 96 weeks, respectively [18]. To our knowledge, no one has, so far, analyzed the effectiveness of TAF/FTC/BIC in meta-analysis studies. In a real-world cohort of virologically suppressed TE PLWH, the maintenance of virological suppression after switching to TAF/FTC/BIC was 97.3% after 12 months of follow-up [33].

In addition, our real-world data confirm the results of the clinical trials. In the TANGO trial, 86% of the patients maintained virological suppression at 144 weeks after switching to DTG/3TC, 93% at 48 weeks, and 86% at 96 weeks. The rate of virological suppression at 144 weeks of TAF-based 3- or 4-DRs was 82% [15], 93% at 48 weeks, and 79% at 96 weeks. In the SALSA study, the virological suppression rates at 48 weeks in the DTG/3TC group versus the current 3-/4-drug antiretroviral regimens were 94% and 93%, respectively [16].

In this study, LLV was detected in 1.8% of the 2-DR group versus 0.9% of the 3-DR group in a follow-up of about 2 years. These results differed slightly from those reported by Gan et al., who documented an incidence of LLV of 4.9% and 3.6% in the DTG/3TC and TAF/FTC/BIC groups at 48 weeks, respectively [37]. Blip occurred mainly in the 3-DR group, while in a Spanish real-word cohort, blip was similar in both groups (around 11%) [38].

Interestingly, no virological failure was observed in both groups. Real-world data presented by Knobel et al. also reported that VF was infrequent for both regimens (1.1% for the DTG/3TC cohort and 0.9% for the TAF/FTC/BIC cohort) and was associated with suboptimal treatment adherence [38]. Similarly, a meta-analysis by Punekar et al. described that only 1% of PLWH on DTG/3TC resulted in VF [26]. For TAF/FTC/BIC, data from the Italian Cohort of Antiretroviral Naïve PLWH (ICONA) group showed a VF of 0.7% at 12 months of follow-up [34].

Most of the PLWH remained on therapy in both groups. Treatment discontinuations were lower than 10% in our cohort and no significant differences were observed between the two groups analyzed. These findings confirm those of other real-life studies, such as Rocabert et al., who described a discontinuation rate for any reason of 7% in the TAF/FTC/BIC group for 1029.30 person years and 8% in the DTG/3TC group for 635.33 person years [36], and Knobel et al., who reported a discontinuation rate of 15.4% with a total follow-up of 815.99 person years for the TAF/FTC/BIC group and 8.7% for 704.37 person years for the DTG/3TC group [37]. In this study, although PLWH in the 3-DR group showed more discontinuations, the event was mainly determined by simplification to dual therapy, which was linked to the physician’s choice. Among the regimen discontinuation episodes due to AEs, four PLWH (3.6%) on the 2-DR and five (2.3%) on the 3-DR were involved. The real-world data from Knobel et al. reported a similar discontinuation rate due to AEs in both groups (1.9% on DTG/3TC and 2.1% on TAF/FTC/BIC) [38]. Interestingly, in this study, the most represented etiology was musculoskeletal, followed by neurological in both groups, and renal and cutaneous in the 3-DR group. These findings are different from those reported in clinical trials [6,7,11,12,15,16,39,40,41], previous observational studies [10,43,44,45], and real-life cohorts [36,37], which showed that neuropsychiatric adverse events (NPAEs) and gastrointestinal toxicity were the main cause of discontinuation of INIs. In a meta-analysis, Pérez-Valero et al. reported that TD due to NPAEs was higher for DTG-based regimens than for TAF/FTC/BIC [46]. However, the evidence of TAF/FTC/BIC versus DTG/3TC discontinuation in relation to neuropsychiatric symptoms is inconsistent [32,35,47].

The results from TANGO RCTs showed that the safety profile of the DTG/3TC regimen was similar to that of the TAF-based arm. Adverse events leading to withdrawal occurred in 4% of PLWH 144 weeks after switching to DTG/3TC and in 1% on TAF-based regimens, and these rates were comparable after 48 weeks [15]. In a large real-world cohort, NPAEs were similar in both the TAF/FTC/BIC and DTG/3TC groups and led to treatment discontinuation in a small proportion of PLWH [36].

Regarding the lipid profile, both regimens showed a similar favorable effect on triglycerides and total cholesterol parameters. These results are in line with those obtained by Baldin et al., which highlighted these findings in relation to the negative impact on the metabolic profile of TAF-based regimens described in the literature [35]. The results are also in line with Rocabert et al., who reported a significant decrease in lipids in both the DTG/3TC and TAF/FTC/BIC groups, without differences between regimens [36].

Data from the RUMBA study demonstrated favorable changes in HDL cholesterol and the impact on metabolic outcomes of DTG/3TC compared to TAF/FTC/BIC at week 48 [48]. However, Heseltine et al.’s study demonstrated an improvement in lipid profiles in PLWH on TAF/FTC/BIC, with the most adverse lipid profiles at the baseline [49]. On the other hand, a general improvement in lipid parameters was already reported over time with the second-generation INI therapies [50].

Notably, in this study, in both cohorts, no differences in weight gain were observed. This result is in line with the Spanish real-world experience described by Knobel et al. [36]. Nevertheless, some studies have reported concerns about an increase in body weight in PLWH treated with second-generation INIs [51,52,53,54], particularly in combination with TAF [6]. In our study, there was no negative impact on body weight or lipid profile. These results may be relevant in the setting of an optimization strategy, considering the aging of the PLWH and the rates of cardiovascular comorbidities.

In conclusion, the 2-DR maintained virological outcomes and safety, allowing for a reduction in lifetime antiretroviral exposure and the supposed possibility of reducing long-term toxicities, so 2-DRs are considered a good option for switching [14,28]. On the other hand, switching to a 3-DR is generally preferred, considering the restrictions for 2-DRs based on clinical history [7]. In a retrospective study with Trio Health HIV Network EMR data, Sax et al. compared the clinical and sociodemographic baseline characteristics which influenced the switching strategy to DTG/3TC versus TAF/FTC/BIC. The prescription of TAF/FTC/BIC was associated with good immunovirological parameters and adherence (e.g., viral suppression, CD4 < 200 cells/uL, and substance use) [55]. DTG/3TC, instead, was associated with renal toxicity and obesity. Similarly, in our clinical practice, physicians tend to prescribe the 2-DR for PLWH with a better immunological profile and more comorbidities and the 3-DR for PLWH with a worse drug resistance profile and immunological status.

The strength of this study includes its real-world setting, which may be helpful in a tailored INI-based switch strategy in clinical practice; however, this study has several limitations. The retrospective design and the relatively small sample size limited the statistical power of comparing the treatment regimens. Furthermore, a different follow-up time between groups may have influenced the outcome evaluations; this is particularly true for the 3-DR cohort, which had a longer follow-up time and a shorter time of viral suppression prior to switching.

## 5. Conclusions

In this study analyzing virologically suppressed treatment-experienced PLWH, DTG/3TC and TAF/FTC/BIC showed similar good effectiveness and safety profiles. Both optimization strategies showed high tolerability without significant differences. In our clinical practice, we observed that physicians tend to prescribe the 2-DR to PLWH with a better immunological profile and more comorbidities and the 3-DR to PLWH with a worse drug resistance profile and immunological status. Further research is expected to support the currently available results in different subpopulations which are not yet fully investigated; this could help in selecting if PLWH are more suitable for the 2-DR or 3-DR strategy.

## Figures and Tables

**Table 1:** Baseline characteristics of the study population.

Characteristics	3TC/DTG *n* = 110	BIC/FTC/TAF *n* = 214	Total *n* = 324	*p*-Value
Age, years	56.0	56.5	56.3	0.653
Median (IQR)	(44.0–63.0)	(48.0–61.5)	(45.9–61.8)	
Sex at birth, *n* (%)				
Male	82 (74.5%)	172 (80.4%)	254 (78.4%)	0.227
Female	28 (25.5%)	42 (19.6%)	70 (21.6%)	
Nationality, *n* (%)				
Italian	90 (81.8%)	171 (79.9%)	261 (80.6%)	0.681
Non-italian	20 (18.2%)	43 (20.1%)	63 (19.4%)	
Sexual orientation, *n* (%)				
Heterosexual	23 (15%)	57 (37.3%)	80 (52.3%)	0.241
MSM	15 (39.5%)	58 (50.4%)	73 (47.7%)	
BMI, kg/m^2^	25.6	25.7	25.6	0.770
Median (IQR)	(23.4–28.4)	(23.4–28.6)	(23.4–28.5)	
Comorbidities, number Median (IQR)	2 (1.0–5.0)	1 (0–2.0)	1 (0–3.0)	<0.0001
Systemic arterial hypertension	44 (40%)	56 (26.2%)	100 (30.9%)	0.011
Cardiovascular disease	26 (23.6%)	13 (6.1%)	39 (12%)	<0.0001
Dyslipidaemia	70 (63.6%)	65 (30.4%)	135 (41.7%)	<0.0001
Obesity	25 (22.7%)	26 (12.1%)	51 (15.7%)	0.013
Chronic kidney disease	19 (17.3%)	10 (4.7%)	29 (9%)	<0.0001
Diabetes mellitus	15 (13.6%)	26 (12.1%)	41 (12.7%)	0.703
Non-AIDS-defining malignancy	6 (5.5%)	13 (6.1%)	19 (5.9%)	0.822
HBV co-infection, *n* (%)	0 (0%)	7 (3.3%)	7 (2.2%)	0.055
HCV co-infection, *n* (%)	12 (10.9%)	38 (17.8%)	50 (15.4%)	0.106
Prior AIDS defining illness, *n* (%)	22 (20%)	60 (28.2%)	82 (25.4%)	0.110
Nadir CD4+ T-cell count (cells/μL),	297.0	214.0	261.0	0.015
Median (IQR)	(172.0–473.0)	(59.0–365.0)	(70.0–406.0)	
No drug resistance, *n* (%)	95 (94.1%)	102 (65.8%)	197 (77%)	
Total time on previous cART (months),	132.0	120.0	122.0	0.108
Median (IQR)	(72.0–228.0)	(72.0–180.0)	(72.0–192.0)	
Time of virologic suppression prior to switch (months),	98.1	80.5	84.4	0.035
Median (IQR)	(57.0–151.0)	(39.5–138.7)	(45.7–142.2)	
Previous treatments, *n* (%)				
INI	78 (70.9%)	172 (80.4%)	250 (77.2%)	0.055
PI	13 (11.8%)	32 (15.0%)	45 (13.9%)	0.440
NNRTI	26 (23.6%)	14 (6.5%)	40 (12.3%)	<0.0001
TAF-based	45 (40.9%)	157 (73.4%)	202 (62.3%)	<0.0001
ABC-based	50 (45.5%)	23 (10.7%)	73 (22.5%)	<0.0001

MSM: Men who have sex with men; BMI: body mass index; cART: combination antiretroviral therapy; INI: integrase inhibitors, NNRTI: non-nucleoside reverse transcriptase inhibitors; PI: protease inhibitors; TAF: tenofovir alafenamide; ABC: abacavir; IQR: interquartile range.

**Table 2:** Treatment outcomes and laboratory parameters of the study population.

	3TC/DTG *n* = 110	BIC/FTC/TAF *n* = 214	Total *n* = 324	*p*-Value
Time of follow-up, months	19.6	27.5	24.6	0.001
Median (IQR)	(14.2–26.4)	(15.3–32.6)	(14.7–31.4)	
Treatment outcomes, *n* (%) On therapy Discontinuations due to:	103 (93.6%)	193 (90.2%)	296 (91.3%)	0.295
Failure	0 (0%)	0 (0%)	0 (0%)	
Low level viremia	2 (1.8%)	2 (0.9%)	4 (1.2%)	0.495
Blip	5 (4.5%)	25 (11.8%)	30 (9.3%)	0.033
Death	0 (0%)	3 (1.4%)	3 (0.9%)	0.212
Switch	1 (0.9%)	11 (5.1%)	12 (3.7%)	0.056
Adverse events	4 (3.6 %)	5 (2.3%)	9 (2.8%)	0.500
Cutaneous	0 (0%)	1 (20%)	1 (11.1%)	
Neurological	1 (25%)	1 (20%)	2 (22.2%)	
Musculoskeletal	3 (75%)	2 (40%)	4 (44.4%)	
Renal	0 (0%)	1 (20%)	2 (22.2%)	
CD4+ cell count (cells/μL) baseline, *n* (%)	781.5	585.0	662.0	<0.0001
Median (IQR)	(598.0–956.0)	(430.0–841.0)	(491.0–892.0)	
CD4+ cell count (cells/μL) last control, *n* (%)	762	651.0	693.0	0.002
Median (IQR)	(580.0–1014.0)	(473.0–904.0)	(517.0–950.0)	
HIV-RNA ˂ 50 cells/μL last control, *n* (%)	109 (99.1%)	205 (97.2%)	314 (97.8%)	0.260
Delta Triglycerides last control-baseline, mg/dL Median (IQR)	−11 (−48, 13)	−14 (−52, 12)	−13 (−50, 12)	0.775
Delta LDL last control-baseline, mg/dL Median (IQR)	−2.3 (−29.1, 16)	−1 (−22, 15.8)	−1.5 (−23.4, 15.8)	0.682
Delta Cholesterol last control-baseline, mg/dL Median (IQR)	−2 (−36, 20)	−7 (−26, 13)	−6 (−28, 14)	0.767
Delta BMI last control-baseline, kg/m^2^ Median (IQR)	0.0 (0.9, 0.8)	0.0 (−0.9, 0,7)	0.0 (−0.9, 0.7)	0.993

LDL: low-density lipoprotein; BMI: body mass index; IQR: interquartile range.

## Data Availability

Data are contained within the article.

## References

[1] Ryom L., De Miguel R., Cotter A.G., Podlekareva D., Beguelin C., Waalewijn H., Arribas J.R., Mallon P.W.G., Marzolini C., Kirk O. (2022). Major revision version 11.0 of the European AIDS Clinical Society Guidelines 2021. HIV Med..

[2] (2023). Panel on Antiretroviral Guidelines for Adults Adolescents Guidelines for the Use of Antiretroviral Agents in Adults Adolescents with HIV. Department of Health and Human Services. https://clinicalinfo.hiv.gov/en/guidelines/adult-and-adolescent-arv.

[3] WHO Consolidated Guidelines on HIV Prevention, Testing, Treatment, Service Delivery and Monitoring: Recommendations for a Public Health Approach. https://www.who.int/publications/i/item/9789240031593.

[4] Gandhi R.T., Bedimo R., Hoy J.F., Landovitz R.J., Smith D.M., Eaton E.F., Lehmann C., Springer S.A., Sax P.E., Thompson M.A. (2022). Antiretroviral drugs for treatment and prevention of HIV infection in adults: 2022 recommendations of the International Antiviral Society-USA Panel. JAMA.

[5] Documento de Consenso de GeSIDA/División de Control de VIH, ITS, Hepatitis Virales y Tuberculosis del Ministerio de Sanidad Respecto al Tratamiento Antirretroviral en Adultos Infectados por el Virus de la Inmunodeficiencia Humana (Actualización enero 2023), GeSIDA 2023. https://gesida-seimc.org/wp-content/uploads/2023/06/Guia_TAR_V12.pdf.

[6] Wohl D.A., Yazdanpanah Y., Baumgarten A., Clarke A., Thompson M.A., Brinson C., Hagins D., Ramgopal M.N., Antinori A., Wei X. (2019). Bictegravir combined with emtricitabine and tenofovir alafenamide versus dolutegravir, abacavir, and lamivudine for initial treatment of HIV-1 infection: Week 96 results from a randomised, double-blind, multicentre, phase 3, non-inferiority trial. Lancet HIV.

[7] Orkin C., DeJesus E., Sax P.E., Arribas J.R., Gupta S.K., Martorell C., Stephens J.L., Stellbrink H.J., Wohl D., Maggiolo F. (2020). Fixed-dose combination bictegravir, emtricitabine, and tenofovir alafenamide versus dolutegravir-containing regimens for initial treatment of HIV-1 infection: Week 144 results from two randomised, double-blind, multicentre, phase 3, non-inferiority trials. Lancet HIV.

[8] Deeks E.D. (2018). Bictegravir/Emtricitabine/Tenofovir Alafenamide: A Review in HIV-1 Infection. Drugs.

[9] Rossetti B., Fabbiani M., Di Carlo D., Incardona F., Abecasis A., Gomes P., Geretti A.M., Seguin-Devaux C., Garcia F., Kaiser R. (2022). EuResist Network, INTEGRATE study group. Effectiveness of integrase strand transfer inhibitors in HIV-infected treatment-experienced individuals across Europe. HIV Med..

[10] Scarsi K.K., Havens J.P., Podany A.T., Avedissian S.N., Fletcher C.V. (2020). HIV-1 Integrase Inhibitors: A Comparative Review of Efficacy and Safety. Drugs.

[11] Cahn P., Sierra Madero J., Arribas J.R., Antinori A., Ortiz R., Clarke A.E., Hung C.C., Rockstroh J.K., Girard P.M., Sievers J. (2022). Three-year durable efficacy of dolutegravir plus lamivudine in antiretroviral therapy–naive adults with HIV-1 infection. AIDS.

[12] Cahn P., Madero J.S., Arribas J.R., Antinori A., Ortiz R., Clarke A.E., Hung C.C., Rockstroh J.K., Girard P.M., Sievers J. (2020). Durable Efficacy of Dolutegravir Plus Lamivudine in Antiretroviral Treatment-Naive Adults With HIV-1 Infection: 96-Week Results From the GEMINI-1 and GEMINI-2 Randomized Clinical Trials. J. Acquir. Immune Defic. Syndr..

[13] Llibre J.M., Pulido F., García F., García Deltoro M., Blanco J.L., Delgado R. (2015). Genetic barrier to resistance for dolutegravir. AIDS Rev..

[14] Gibas K.M., Kelly S.G., Arribas J.R., Cahn P., Orkin C., Daar E.S., Sax P.E., Taiwo B.O. (2022). Two-drug regimens for HIV treatment. Lancet HIV.

[15] Osiyemi O., De Wit S., Ajana F., Bisshop F., Portilla J., Routy J.P., Wyen C., Ait-Khaled M., Leone P., Pappa K.A. (2022). Efficacy and Safety of Switching to Dolutegravir/Lamivudine Versus Continuing a Tenofovir Alafenamide-Based 3- or 4-Drug Regimen for Maintenance of Virologic Suppression in Adults Living With Human Immunodeficiency Virus Type 1: Results Through Week 144 From the Phase 3, Noninferiority TANGO Randomized Trial. Clin. Infect. Dis..

[16] Llibre J.M., Brites C., Cheng C.Y., Osiyemi O., Galera C., Hocqueloux L., Maggiolo F., Degen O., Taylor S., Blair E. (2023). Efficacy and Safety of Switching to the 2-Drug Regimen Dolutegravir/Lamivudine versus Continuing a 3- or 4-Drug Regimen for Maintaining Virologic Suppression in Adults Living With Human Immunodeficiency Virus 1 (HIV-1): Week 48 Results From the Phase 3, Noninferiority SALSA Randomized Trial. Clin. Infect. Dis..

[17] Martínez-Serra A., De Lazzari E., Berrocal L., Foncillas A., De La Mora L., Inciarte A., Chivite I., González-Cordón A., Martínez-Rebollar M., Torres B. (2023). Clinical use and effectiveness of dolutegravir and lamivudine: A long-term, real-world, retrospective study. J. Antimicrob. Chemother..

[18] Patel R., Evitt L., Mariolis I., Di Giambenedetto S., d’Arminio Monforte A., Casado J., Cabello Úbeda A., Hocqueloux L., Allavena C., Barber T. (2021). HIV Treatment with the Two-Drug Regimen Dolutegravir Plus Lamivudine in Real-world Clinical Practice: A Systematic Literature Review. Infect. Dis. Ther..

[19] Bowman C., Ambrose A., Kanitkar T., Flores K., Simoes P., Hart J., Hunter A., Akodu J., Barber T.J. (2023). Real world use of dolutegravir two drug regimens. AIDS.

[20] Suárez-García I., Alejos B., Hernando V., Viñuela L., Vera García M., Rial-Crestelo D., Pérez Elías M.J., Albendín Iglesias H., Peraire J., Tiraboschi J. (2023). Effectiveness and tolerability of dolutegravir/lamivudine for the treatment of HIV-1 infection in clinical practice. J. Antimicrob. Chemother..

[21] Yang X., Fu Y., Xie X., Gan L., Song C., Song Y., Li J., Long H. (2022). Real-world implementation of dolutegravir plus lamivudine in people living with HIV in Southwest China. Expert Rev. Anti-Infect. Ther..

[22] Cento V., Perno C.F. (2021). Dolutegravir Plus Lamivudine Two-Drug Regimen: Safety, Efficacy and Diagnostic Considerations for Its Use in Real-Life Clinical Practice-A Refined Approach in the COVID-19 Era. Diagnostics.

[23] Lee K.H., Kim J., Lee J.A., Kim C.H., Ahn J.Y., Jeong S.J., Ku N.S., Choi J.Y., Yeom J.S., Song Y.G. (2022). Real-World Effectiveness, Tolerability, and Safety of Dolutegravir/Lamivudine in Korea. Viruses.

[24] Buzón L., Dueñas C., Pedrero R., Iribarren J.A., de Los Santos I., Díaz de Santiago A., Morán M.Á., Pousada G., Moreno E., Ferreira E. (2023). Dolutegravir Plus 3TC in Virologically Suppressed PLWHIV: Immunological Outcomes in a Multicenter Retrospective Cohort in Spain during the COVID-19 Pandemic. Viruses.

[25] Borghetti A., Baldin G., Lombardi F., Ciccullo A., Capetti A., Rusconi S., Sterrantino G., Latini A., Cossu M.V., Gagliardini R. (2018). Efficacy and tolerability of lamivudine plus dolutegravir as a switch strategy in a multicentre cohort of patients with suppressed HIV-1 replication. HIV Med..

[26] Punekar Y.S., Parks D., Joshi M., Kaur S., Evitt L., Chounta V., Radford M., Jha D., Ferrante S., Sharma S. (2021). Effectiveness and safety of dolutegravir two-drug regimens in virologically suppressed people living with HIV: A systematic literature review and meta-analysis of real-world evidence. HIV Med..

[27] Santoro M.M., Armenia D., Teyssou E., Santos J.R., Charpentier C., Lambert-Niclot S., Antinori A., Katlama C., Descamps D., Perno C.F. (2022). Virological efficacy of switch to DTG plus 3TC in a retrospective observational cohort of suppressed HIV-1 patients with or without past M184V: The LAMRES study. J. Glob. Antimicrob. Resist..

[28] Maggiolo F., Gulminetti R., Pagnucco L., Digaetano M., Cervo A., Valenti D., Callegaro A., Mussini C. (2022). Long-term outcome of lamivudine/dolutegravir dual therapy in HIV-infected, virologically suppressed patients. BMC Infect Dis..

[29] Rolle C.P., Nguyen V., Patel K., Cruz D., DeJesus E., Hinestrosa F. (2021). Real-world efficacy and safety of switching to bictegravir/emtricitabine/tenofovir alafenamide in older people living with HIV. Medicine.

[30] Nasreddine R., Florence E., Yombi J.C., Henrard S., Darcis G., Van Praet J., Vandekerckhove L., Allard S.D., Demeester R., Messiaen P. (2023). Efficacy, durability, and tolerability of bictegravir/emtricitabine/tenofovir alafenamide for the treatment of HIV in a real-world setting in Belgium. HIV Med..

[31] Tsai M.S., Sun H.Y., Chen C.P., Lee C.H., Lee C.Y., Liu C.E., Tang H.J., Hung T.C., Li C.W., Lee Y.T. (2023). Switching to coformulated bictegravir, emtricitabine, and tenofovir alafenamide maintained viral suppression in adults with historical virological failures and K65N/R mutation. Int. J. Infect. Dis..

[32] Hoffmann C., Schewe K., Fenske S., Buhk T., Sabranski M., Adam A., Hansen S., Stellbrink H.J. (2020). Short-term neuropsychiatric tolerability of bictegravir combined with emtricitabine/tenofovir alafenamide in clinical practice. Antivir Ther..

[33] Chang H.M., Chou P.Y., Chou C.H., Tsai H.C. (2021). Outcomes After Switching to BIC/FTC/TAF in Patients with Virological Failure to Protease Inhibitors or Non-Nucleoside Reverse Transcriptase Inhibitors: A Real-World Cohort Study. Infect Drug Resist..

[34] D’Arminio Monforte A., Tavelli A., Cingolani A., Taramasso L., Mussini C., Piconi S., Calcagno A., Orofino G., Cicalini S., Castagna A. Effectiveness of bictregravir/emtricitabine/tenofovir alafenamide (BIC/FTC/TAF) as switch strategy in virologically suppressed: Real-world data from the ICONA cohort. Proceedings of the HIV Glasgow 2022.

[35] Baldin G., Ciccullo A., Lombardi F., D’Angelillo A., Dusina A., Emiliozzi A., Farinacci D., Moschese D., Picarelli C., Borghetti A. (2021). Short Communication: Comparing Lamivudine+Dolutegravir and Bictegravir/Emtricitabine/Tenofovir Alafenamide as Switch Strategies: Preliminary Results from Clinical Practice. AIDS Res. Hum. Retroviruses.

[36] Rocabert A., Borjabad B., Berrocal L., Blanch J., Inciarte A., Chivite I., Gonzalez-Cordon A., Torres B., Ambrosioni J., Martinez-Rebollar M. (2023). Tolerability of bictegravir/tenofovir alafenamide/emtricitabine versus dolutegravir/lamivudine as maintenance therapy in a real-life setting. J. Antimicrob. Chemother..

[37] Gan L., Xie X., Fu Y., Yang X., Ma S., Kong L., Song C., Song Y., Ren T., Long H. (2023). Bictegravir/Emtricitabine/Tenofovir Alafenamide Versus Dolutegravir Plus Lamivudine for Switch Therapy in Patients with HIV-1 Infection: A Real-World Cohort Study. Infect Dis. Ther..

[38] Knobel H., Cañas-Ruano E., Guelar A., Knobel P., Villar-García J., González-Mena A., Canepa C., Arrieta-Aldea I., Marcos A., Abalat-Torrres A. (2023). Switching to Dolutegravir/lamivudine or Bictegravir/Emtricitabine/Tenofovir alafenamide. A comparative real-world study. HIV Res. Clin. Pract..

[39] Molina J.M., Ward D., Brar I., Mills A., Stellbrink H.J., López-Cortés L., Ruane P., Podzamczer D., Brinson C., Custodio J. (2018). Switching to fixed-dose bictegravir, emtricitabine, and tenofovir alafenamide from dolutegravir plus abacavir and lamivudine in virologically suppressed adults with HIV-1: 48 week results of a randomised, double-blind, multicentre, active-controlled, phase 3, non-inferiority trial. Lancet HIV.

[40] Daar E.S., DeJesus E., Ruane P., Crofoot G., Oguchi G., Creticos C., Rockstroh J.K., Molina J.M., Koenig E., Liu Y.P. (2018). Efficacy and safety of switching to fixed-dose bictegravir, emtricitabine, and tenofovir alafenamide from boosted protease inhibitor-based regimens in virologically suppressed adults with HIV-1: 48 week results of a randomised, open-label, multicentre, phase 3, non-inferiority trial. Lancet HIV.

[41] Kityo C., Hagins D., Koenig E., Avihingsanon A., Chetchotisakd P., Supparatpinyo K., Gankina N., Pokrovsky V., Voronin E., Stephens J.L. (2019). Switching to Fixed-Dose Bictegravir, Emtricitabine, and Tenofovir Alafenamide (B/F/TAF) in Virologically Suppressed HIV-1 Infected Women: A Randomized, Open-Label, Multicenter, Active-Controlled, Phase 3, Noninferiority Trial. J. Acquir. Immune Defic. Syndr..

[42] Tang M.W., Liu T.F., Shafer R.W. (2012). The HIVdb system for HIV-1 genotypic resistance interpretation. Intervirology.

[43] Hidalgo-Tenorio C., Cortés L.L., Gutiérrez A., Santos J., Omar M., Gálvez C., Sequera S., Jesús S.E., Téllez F., Fernández E. (2019). DOLAMA study: Effectiveness, safety and pharmacoeconomic analysis of dual therapy with dolutegravir and lamivudine in virologically suppressed HIV-1 patients. Medicine.

[44] Joly V., Burdet C., Landman R., Vigan M., Charpentier C., Katlama C., Cabié A., Benalycherif A., Peytavin G., Yeni P. (2019). Dolutegravir and lamivudine maintenance therapy in HIV-1 virologically suppressed patients: Results of the ANRS 167 trial (LAMIDOL). J. Antimicrob. Chemother..

[45] Wandeler G., Buzzi M., Anderegg N., Sculier D., Béguelin C., Egger M., Calmy A. (2018). Virologic failure and HIV drug resistance on simplified, dolutegravir-based maintenance therapy: Systematic review and meta-analysis. F1000Research.

[46] Pérez-Valero I., Corona D., Martínez N., López-Cavanillas M., Lluis C., Luque I. (2023). Real-world discontinuations due to neuropsychiatric symptoms in people living with HIV treated with second-generation integrase inhibitors: A systematic review. Expert Rev. Anti-Infect. Ther..

[47] Hoffmann C., Llibre J.M. (2019). Neuropsychiatric adverse events with dolutegravir and other integrase strand transfer inhibitors. AIDS Rev..

[48] DeGroote S., Vanherrewage S., Tobback E., Caluwé E., Vincke L., Blomme E., Vandekerckhove L., De Scheerder M.A. Understanding changes in metabolic parameters switching to 2DR from 3DR Integrase Strand Inhibitors (InSTIs). Proceedings of the HIV Glasgow 2022.

[49] Heseltine T., Hughes E., Mathew J., Murray S., Khoo S. (2022). The effect of changing to Bictegravir on lipids using real world data: A brief report. J. Clin. Pharm. Ther..

[50] Taramasso L., Bonfanti P., Ricci E., Maggi P., Orofino G., Squillace N., Menzaghi B., Madeddu G., Molteni C., Vichi F. (2022). Metabolic syndrome and body weight in people living with HIV infection: Analysis of differences observed in three different cohort studies over a decade. HIV Med..

[51] Bai R., Lv S., Wu H., Dai L. (2022). Effects of different integrase strand transfer inhibitors on body weight in patients with HIV/AIDS: A network meta-analysis. BMC Infect Dis..

[52] Hester E.K., Greenlee S., Durham S.H. (2022). Weight Changes With Integrase Strand Transfer Inhibitor Therapy in the Management of HIV Infection: A Systematic Review. Ann. Pharmacother..

[53] Bansi-Matharu L., Phillips A., Oprea C., Grabmeier-Pfistershammer K., Günthard H.F., De Wit S., Guaraldi G., Vehreschild J.J., Wit F., Law M. (2021). Contemporary antiretrovirals and body-mass index: A prospective study of the RESPOND cohort consortium. Lancet HIV.

[54] Kileel E.M., Malvestutto C.D., Lo J., Fitch K.V., Fichtenbaum C.J., Aberg J.A., Zanni M.V., Martinez E., Okeke N.L., Kumar P. (2023). Changes in body mass index with Longer-term Integrase Inhibitor Use: A Longitudinal Analysis of integrase inhibitors: A longitudinal analysis of data from the randomised trial to Prevent Vascular Events in Human Immunodeficiency Virus (REPRIEVE). Clin. Infect. Dis..

[55] Sax P.E., JEron J., Radtchenko J., Dunbar M., JGruber J., Fridman M., Ramgopal M., Mounzer K., Huhn G., Santiago S. What influences switching to DTG/3TC vs. B/F/TAF in clinical practice?. Proceedings of the CROI 2023.

